# Parental Practices and Attitudes Related to Smoke-Free Rules in Homes, Cars, and Outdoor Playgrounds in US Households With Underage Children and Smokers, 2010–2011

**DOI:** 10.5888/pcd12.140553

**Published:** 2015-06-18

**Authors:** Xiao Zhang, Ana Martinez-Donate, Natalie Rhoads

**Affiliations:** Author Affiliations: Xiao Zhang, Natalie Rhoads, Department of Population Health Sciences, University of Wisconsin-Madison, Madison, Wisconsin.

## Abstract

**Introduction:**

A smoke-free environment protects children from exposure to involuntary smoke and also can reduce or prevent future smoking behavior. The purpose of this study was to examine levels and correlates of parental behavior and attitudes related to voluntary smoke-free rules in homes, cars, and outdoor children’s play areas among US households with underage children and 1 or more smoking parents.

**Methods:**

We used data from the 2010–2011 Tobacco Use Supplement to the Current Population Survey and logistic regressions to model behavior and attitudes related to voluntary smoke-free rules in 3 settings.

**Results:**

Overall, 60.1% of households with children and at least 1 smoking parent had voluntary smoke-free home rules. Approximately 84.6% and 71.5% of parents thought that smoking should not be allowed inside cars with children present and in outdoor play areas, respectively. Positive parental behavior and attitudes related to voluntary smoke-free rules were more likely among households with 2 parents, parents of higher education and household income, Hispanic parents, and parents of infants (*P* < .05).

**Conclusion:**

Tobacco control and prevention efforts are needed to promote the voluntary adoption of smoke-free rules in homes, private cars, and outdoor children’s play areas. Most parents from smoker households with underage children were supportive of smoke-free laws for cars and outdoor children’s play areas, providing evidence and encouragement to policy makers to take action to restrict smoking in these locations.

## Introduction

Children living with 1 or more smoking parents are at increased risk for involuntary smoke exposure, which can cause many negative health outcomes (1). Children living with smokers are also more likely to smoke, even after accounting for other sociodemographic factors (2,3). A smoke-free environment can not only protect children from exposure to involuntary smoke, but also convey an antitobacco social norm that prevents and reduces smoking behavior in the future (4–7). The home, the car, and children’s outdoor play areas are 3 primary sources of involuntary smoke exposure for children (8).

Half of US households with underage children and at least 1 smoking parent did not have a voluntary smoke-free home rule by 2007, versus more than 90% of households with children but no smoking parents (9). However, it is unclear what the prevalence has become in recent years. Furthermore, research has examined voluntary smoke-free rules in cars (10–12) and has found overwhelming support among the US general population for voluntary smoke-free car policies when children are present (13). Yet, support for voluntary car rules has not been examined among households with children and at least 1 smoking parent, a vulnerable subset of households. Even less is known about attitudes toward smoking restrictions in outdoor children’s play areas like playgrounds and sport fields.

The 2010–2011 Tobacco Use Supplement to the Current Population Survey (TUS-CPS) collected information on respondents’ reports of voluntary smoke-free home rules and attitudes related to smoking restrictions in cars and outdoor children’s play areas. Attitudes are theoretical determinants and good predictors of behaviors (14) and also reflect levels of support for future legislation to expand public smoke-free laws. Using data from this survey, we aimed to update the prevalence of voluntary smoke-free home rules and examine the distribution of parental attitudes related to voluntary smoke-free rules in cars and outdoor children’s play areas among US households with underage children and 1 or more smoking parents during 2010–2011. We also investigated parental and household factors associated with these outcomes.

## Methods

### Study population

We used data from the 2010–2011 TUS-CPS. The TUS-CPS is a survey of tobacco use that is administered as part of the US Census Bureau and the Bureau of Labor Statistics’ CPS. A large sample of households is surveyed during a survey period that provides representative data on tobacco-related behaviors, norms, and attitudes at the national and state levels among the US civilian noninstitutionalized population. All permanent household members aged 18 years or older are eligible for the interview. We included only primary family households with underage children (younger than 18) and at least 1 parent who smoked in our analysis. We defined a parent as a household’s reference person (head of the household) or his or her spouse, which means this person may not be the child’s biological parent. Some items from the TUS-CPS may be self-reported or provided by proxy (eg, smoking status and the use of other tobacco products), whereas the practices and attitudes related to voluntary smoke-free rules in home, car, and outdoor play areas were only self-reported. Therefore, we limited our study sample to respondents who provided self-reported data.

### Measures

We defined a smoking parent as the reference person of a household or his or her spouse who had smoked 100 cigarettes or more and was smoking every day or some days at the time of the interview (15).

All self-respondents of the TUS-CPS were asked “Which statement best describes the rules about smoking inside your home?” We categorized the report of “No one is allowed to smoke anywhere inside your home” as a voluntary smoke-free home rule. A smoke-free household is a single-parent household in which the parent reports a voluntary smoke-free home rule or a 2-parent household in which at least 1 parent reports voluntary smoke-free home rules. A previous study indicated that discordance between parental reports of home rule decreased over time and has dropped to below 5% among 2-parent households with children and at least 1 smoking parent by 2007 (16), making the use of a single parental report reliable.

Parents were asked, “Inside a car, when there are other people present, do you think that smoking should always be allowed, be allowed under some conditions, or never be allowed?” Those selecting the first 2 options were further asked, “If children are present inside the car, do you think that smoking should always be allowed, be allowed under some conditions, or never be allowed?” We defined a positive attitude toward a voluntary smoke-free car rule when children are present as the report of smoking should never be allowed when there are other people present or if children are present inside the car. Hereafter, this attitude is referred to as support for a voluntary smoke-free car rule.

The TUS-CPS asked respondents “On outdoor children’s playgrounds and outdoor children’s sport fields, do you think that smoking should be allowed in all areas, allowed in some areas, or not allowed at all?” The response “not allowed at all” was defined as an indicator of support for a rule for voluntary smoke-free outdoor children’s play areas.

We analyzed the following parental and household factors potentially associated with the establishment of smoking restrictions: household structure, highest parental education level, race/ethnicity, age, smoking status, age of the youngest child, and annual household income. We also included state identifiers to capture unmeasured state-level factors, such as statewide tobacco control policies, which may be associated with the outcomes.

### Statistical analysis

We calculated descriptive statistics on the prevalence of parental practice and attitudes regarding smoke-free rules in the home, the car, and outdoor children’s play areas. We conducted multivariate logistic regressions to examine associated parental and household factors with the report of smoke-free rules. For the existence of smoke-free home rules, the analyses were performed on the household level; for attitudes toward smoke-free rules in the car and outdoor children’s play areas, the analyses were performed on the individual level. Survey weights provided by the TUS-CPS were used, and clustered standard errors were estimated to account for the complex sampling design and clustering to produce population estimates. We performed all analyses using the software STATA/MP version 13.0 (StataCorp LP), and significance was set at *P* < .05.

## Results

The overall response rate for the 2010–2011 TUS-CPS was 74% (ie, 74% of eligible individuals responded to the supplement). The rate for self-respondents was 56% (ie, 56% of eligible individuals answered the supplement themselves). Our study sample included parents from 8,083 households, of which 37.8% were single-parent households and 62.2% 2-parent households (Table 1).

**Table 1:** Parental and Household Characteristics, Households With Underage Children and 1 or More Smoking Parents (N = 8,083), United States^a^, 2010–2011

Parental or Household Characteristic	All Households, %
**Household structure**	
Single-parent	37.8
2-Parent	62.2
**Highest education level**	
Less than high school	12.3
High school graduate	70.8
College graduate	16.9
**Age of youngest child, y**	
<1	8.8
1–5	37.6
6–12	33.0
≥13	20.6
**Annual household income, $**	
<25,000	33.9
25,000–49,999	30.3
≥50,000	35.8
**Reference parent’s race/ethnicity**	
Non-Hispanic white	69.7
Non-Hispanic African American	11.5
Hispanic	13.2
Other	5.7
**Reference parent’s age, y**	
18–29	20.9
30–39	34.5
40–49	29.2
≥50	15.4

a Analyses were conducted at the household level.

Overall, 60.1% (95% confidence interval [CI], 58.8%–61.3%) of households with children and 1 or more smoking parent established a voluntary smoke-free home rule inside the home. Most (84.6%; 95% CI, 83.8%–85.4%) parents from these households thought that smoking should never be allowed inside cars when children are present, including 72.2% of those who did not have a voluntary smoke-free rule in their homes (Figure). Fewer parents (71.5%; 95% CI, 70.5%–72.5%) were supportive of smoke-free rules in outdoor children’s play areas, which included 61.3% of those not from a smoke-free household.

**Figure:**
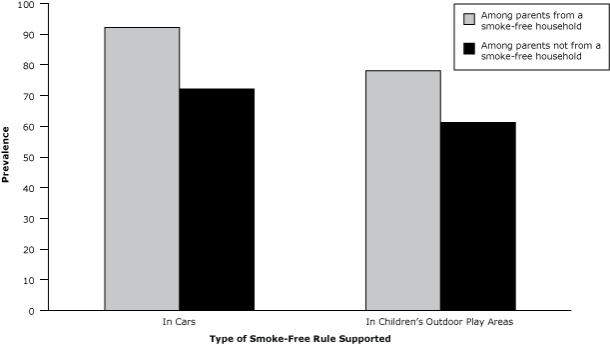
Support for voluntary smoke-free rules in cars and outdoor children’s play areas among parents from and not from smoke-free households, United States, 2010–2011. A smoke-free household is a single-parent household in which the parent reports a voluntary smoke-free home rule or a 2-parent household in which at least 1 parent reports voluntary smoke-free home rules. **Table:** 

Type of Voluntary Smoke-Free Rule Supported	Parents From a Smoke-Free Household, %	Parents Not From a Smoke-Free Household, %
Car	92.2	72.2
Outdoor area	78.1	61.3

On the household level, children whose parents were single fathers or mothers, had not completed high school education, were non-Hispanic African American, or were aged 40 years or older were less likely to be protected by a voluntary smoke-free home rule than were their counterparts (*P* < .05) (Table 2). Households with an annual income less than $25,000 and with a child aged 6 years or older were also less likely to institute a voluntary smoke-free home rule (*P* < .05). 

**Table 2:** Parental and Household Factors Associated With Existence of Smoke-Free Home Rules^a^ Among Households With Children and 1 or More Smoking Parents (N = 8,083)^b^, 2010–2011

Parental or Household Factor	Adjusted Odds Ratio (95% Confidence Interval)
**Household structure**	
Single-parent	1 [Reference]
2-Parent	1.45 (1.26–1.62)
**Highest education level**	
Less than high school	1 [Reference]
High school graduate	1.34 (1.12–1.60)
College graduate	2.73 (2.14–3.48)
**Age of youngest child, y**	
<1	1 [Reference]
1–5	0.85 (0.67–1.06)
6–12	0.54 (0.43–0.67)
≥13	0.42 (0.32–0.53)
**Annual household income, $**	
<25,000	1 [Reference]
25,000–49,999	1.21 (1.05–1.39)
≥50,000	2.00 (1.71–2.34)
**Reference parent’s race/ethnicity**	
Non-Hispanic white	1 [Reference]
Non-Hispanic African American	0.63 (0.52–0.77)
Hispanic	2.13 (1.72–2.64)
Other	1.19 (0.91–1.55)
**Reference parent’s age, y**	
18–29	1 [Reference]
30–39	0.90 (0.76–1.07)
40–49	0.66 (0.55–0.80)
≥50	0.50 (0.40–0.61)

a A smoke-free home is a single-parent household in which the parent reported a smoke-free home rule or a 2-parent household in which at least 1 parent reported a smoke-free rule.

b Analyses were conducted at the household level and adjusted for state of residence.

A positive attitude toward a voluntary smoke-free car rule when children are present was more likely to be reported by individuals from 2-parent households, who had a college education, who were non-Hispanic African American or Hispanic, with an annual household income of $50,000 or more, and who had infants living in the home (*P* < .05) (Table 3). Similar predictors were observed for a supportive attitude toward smoke-free outdoor children’s play areas. Compared with non-Hispanic white parents, non-Hispanic African American and Hispanic parents were more likely to express that smoking should be banned in outdoor children’s play areas (*P* < .05). The attitude was also more likely to be reported by people from 2-parent households than by those from single-parent households and by those who had a college degree than by those who did not (*P* < .05). In additional analyses, we found that support for smoke-free rules was stronger from nonsmoker households with children; 96.3% of parents from these households advocated voluntary smoke-free rules in cars, and 87.4% of parents from these households advocated voluntary smoke-free rules in outdoor areas (data not shown).

**Table 3:** Parental and Household Factors Associated With Supportive Parental Attitudes Toward Smoke-Free Rules in Cars and Outdoor Children’s Areas, Households With Children and 1 or More Smoking Parents (N = 13,111)^a^, 2010–2011

Parental or Household Factor	Cars, AOR (95% CI)	Outdoor Children’s Areas, AOR (95% CI)
**Household structure**		
Single-parent	1 [Reference]	
2-Parent	1.45 (1.25–1.68)	1.19 (1.04–1.36)
**Education level**		
Less than high school	1 [Reference]	
High school graduate	1.06 (0.89–1.26)	1.06 (0.90–1.25)
College graduate	1.87 (1.42–2.47)	1.50 (1.16–1.94)
**Age of youngest child, y**		
<1	1 [Reference]	
1–5	0.87 (0.66–1.13)	0.95 (0.75–1.19)
6–12	0.64 (0.48–0.84)	1.03 (0.81–1.31)
≥13	0.64 (0.48–0.87)	1.00 (0.77–1.30)
**Annual household income, $**		
<25,000	1 [Reference]	
25,000–49,999	0.98 (0.83–1.14)	0.93 (0.80–1.09)
≥50,000	1.38 (1.15–1.65)	1.03 (0.76–1.23)
**Race/ethnicity**		
Non-Hispanic white	1 [Reference]	
Non-Hispanic African American	2.05 (1.59–2.64)	1.49 (1.20–1.84)
Hispanic	3.20 (2.33–4.39)	1.95 (1.54–2.46)
Other	1.31 (0.96–1.79)	0.98 (0.75–1.28)
**Age, y**		
18–29	1 [Reference]	
30–39	0.96 (0.79–1.16)	1.05 (0.88–1.25)
40–49	0.80 (0.65–0.99)	0.90 (0.74–1.10)
≥50	0.85 (0.66–1.08)	0.71 (0.57–0.88)

Abbreviations: AOR, adjusted odds ratio; CI, confidence interval.

a Analyses were conducted at the individual level and adjusted for state of residence and household cluster effects.

## Discussion

We found that more than one-third of US households with children and 1 or more smoking parents had not voluntarily adopted smoke-free home rules by 2010–2011. Children living in these homes are likely to be exposed to involuntary smoke. The existence of a voluntary smoke-free home rule was associated with parental education, parental race/ethnicity, parental age, household income, age of the youngest child, and household structure. The results are in agreement with those of other studies that found persistent disparities by these parental and household factors (9,17–20), and indicate that disparities in home rules have not improved by 2010–2011. Healthy People 2020 calls for increasing the overall proportion of voluntary smoke-free homes by approximately 10% (from 79.1% to 87.0%) and eliminating health disparities (21). Therefore, continued work is warranted to encourage the adoption of voluntary smoke-free home rules and reduce disparities in involuntary smoke exposure and tobacco-use–related diseases among smoker households with children.

Most parents agreed that smoking should not be allowed in cars when children are present. Strong support for voluntary smoke-free rules in cars may reflect the perception that the space in a car is more confined than a home and suggests that parents are more concerned about involuntary smoke exposure for children in a car. Parents may perceive that it is safe to smoke in certain areas of the home (eg, different rooms, balconies) or at certain times, as long as children are not present. In comparison, a car offers less ambiguity about likely exposure of children riding with smoking adults. In addition, time spent in a car is usually short and requires less effort from parents to refrain from smoking. More than two-thirds of parents thought smoking should be disallowed in outdoor play areas, which was not as strong a preference as that for car rules. However, a preference for voluntary smoke-free rules in outdoor areas could indicate more antitobacco attitudes, considering that the concentration of involuntary smoke in an outdoor environment may be lower than that of indoor settings (22). These results suggest widespread support among parents for comprehensive legislation banning smoking in areas where children are likely to be present. The support in states with strong antitobacco programs, such as California, Florida, and Massachusetts, is generally higher than the national benchmark (Appendix).

Attitudes are likely to overestimate actual parental behaviors regarding rules restricting smoking voluntarily in cars and outdoor children’s play areas. For example, Cheng et al found that at the national level the rates of voluntary smoke-free rules in home and cars among US households with smokers were similar (20). However, in our study, the rate of support for voluntary car rules was substantially higher than the rate of existence of voluntary home rules. According to the theory of planned behavior — which posits that personal attitudes toward behavior, subjective norms (social pressure), and perceived behavioral control together shape the behavioral intentions and behaviors of an individual (14) — the gap between attitudes and behaviors may be explained by lack of perceived control over the behavior and subjective norms. For example, compared with parents in 2-parent households, single parents may be limited in their ability to step away from children to smoke outside the home. Likewise, smoking parents may be more likely to be surrounded by others who smoke and this, in turn, may dissuade them from implementing voluntary smoke-free rules in their homes and cars. The high rates of positive attitudes, however, suggest that smoking parents are aware of the harm of smoking around children and possess high level of readiness to implement voluntary smoke-free rules in cars and outdoor play areas.

Our findings have implications for public health efforts, including policies aiming to help parents overcome barriers and translate their antitobacco beliefs and attitudes into actions. Health promotion programs should emphasize the threat of involuntary smoke exposure to children and promote the voluntary adoption of smoke-free rules in homes and private cars. Efforts to encourage (eg, through campaigns directed to resident associations and landlords) or even mandate smoking bans in outdoor areas in housing complexes should be implemented. A “complete” voluntary smoke-free car rule when children are present should also be recommended or even required by law, as some states and municipalities have already done. Because residual tobacco smoke contamination that remains after the cigarette is extinguished (known as thirdhand smoke) can continue to harm the health of children (23,24), public education about thirdhand smoke and its detrimental health effects should be increased to promote adoption of voluntary smoking bans, even when children are not present.

The widespread endorsement of negative attitudes about smoking in cars and outdoor areas in smoker households is notable, considering smokers traditionally hold less negative views about tobacco and are less likely to have a home nonsmoking rule (9). Our additional analyses showed that support for nonsmoking rules is even greater among nonsmoker households with children. Combined, these results suggest that most parents in households with underage children, regardless of smoking status, would be supportive of legislation to ban smoking in these areas. Most provinces of Canada and all states of Australia have now prohibited smoking in motor vehicles with a minor present (25). In the United States, 7 states (Arkansas, California, Louisiana, Maine, Oregon, Utah, and Vermont) implemented statewide policies to ban smoking in cars when children are present, although the cutoff age for child passengers varies by state (26). In addition, certain cities or counties in Hawaii, Indiana, New Jersey, and New York also have similar bans. We recommend that all states pass laws to ban smoking in vehicles when there are underage children present. Likewise, policy makers should also consider extending smoke-free policies to outdoor children’s play areas. Across the country, California has led the way and mandated smoke-free playground spaces designated for children (27). Its experience should inform similar efforts in other states.

Attitudes toward voluntary smoke-free rules in car and outdoor play areas were predicted by parental and household factors similar to voluntary home rules. We found that, compared with non-Hispanic white parents, African American parents were less likely to voluntarily adopt a smoke-free home rule, but they were more likely to report a positive attitude toward voluntary smoke-free rules in cars and outdoor play areas. There is no reason to believe that potential information bias is systematically different between the report of attitudes and behaviors. Therefore, such findings are suggestive that African American smoking parents may encounter special difficulties in setting up voluntary smoke-free rules in the home. For example, they may have less access to other adults who can watch their children when they step out to smoke. The findings may also reflect that African American families are more likely to live in unsafe neighborhoods that make it challenging to smoke outside the home (28). These results underscore the need to conduct additional research to identify barriers to voluntary adoption of smoking bans among these populations and interventions to support their implementing smoke-free rules in homes, cars, and outdoor play areas.

This study has limitations. First, parental practice and attitudes related to voluntary smoke-free rules are based on self-reported data. As smoking continues to decline in perceived normalcy, these findings may be biased due to social desirability. Second, positive attitudes toward voluntary smoke-free rules in cars and outdoor children’s play areas do not necessarily equal support for legislation in corresponding places. For example, some individuals may think that it is their decision to implement a smoke-free rule in their cars and the government should not interfere and dictate this behavior. Therefore, future surveys should ask respondents about their own behaviors in these places and about their support for legislation applying to these 2 settings. Third, the response rate for self-reported data was moderate and may have caused selection bias. However, the rate is within range (40%–60%) recommended for surveys of high importance about decisions on key policies or resources allocation (29).

We found that children living in more than one-third of households with 1 or more smoking parents were not protected by voluntary smoke-free home rules by 2010–2011. The findings call for tobacco control and prevention efforts to continue to promote voluntary smoke-free home rules among households with both smoking parents and underage children, especially among households with single parents, parents of lower socioeconomic status, and parents without infants. We also found that parents from smoker households are generally supportive of voluntary smoke-free rules in cars when children are present and in outdoor children’s play areas, providing evidence and encouragement to policy makers to take action to ban smoking in these locations.

## Appendix Prevalence of Smoke-Free Home Rules and Parental Supportive Attitudes Toward Smoke-Free Rules in Cars and Outdoor Children’s Play Areas Among Smoker Households With Underage Children, by State, United States, 2010–2011

**Table:**

State	Existence of Smoke-Free Home Rules^a^	Attitudes Toward Smoke-Free Rules in Cars^b^	Attitudes Toward Smoke-Free Rules in Outdoor Children’s Play Areas^b^
	%		
Alabama	42.9	79.1	58.2
Alaska	71.8	87.0	74.6
Arizona	73.0	89.4	73.2
Arkansas	46.7	82.7	61.7
California	84.8	97.2	84.8
Colorado	75.9	90.4	72.3
Connecticut	63.9	89.4	78.3
Delaware	58.1	84.2	70.4
Washington, DC	43.8	89.9	74.0
Florida	68.8	88.6	74.8
Georgia	65.6	86.8	80.2
Hawaii	72.1	90.9	64.9
Idaho	77.0	84.9	74.1
Illinois	48.6	81.6	74.1
Indiana	41.8	80.9	61.3
Iowa	55.2	86.9	74.1
Kansas	49.9	75.8	65.2
Kentucky	42.1	78.6	56.9
Louisiana	64.3	84.9	65.9
Maine	65.4	89.3	85.9
Maryland	61.1	87.1	76.5
Massachusetts	65.5	91.3	82.0
Michigan	54.0	80.6	69.5
Minnesota	66.4	84.4	75.7
Mississippi	55.8	84.0	73.5
Missouri	48.9	80.9	58.3
Montana	59.0	86.6	75.4
Nebraska	63.9	88.1	72.3
Nevada	64.6	89.3	75.0
New Hampshire	61.3	83.8	73.8
New Jersey	68.3	94.0	76.1
New Mexico	64.6	80.0	66.7
New York	57.8	89.9	82.6
North Carolina	47.2	79.1	65.4
North Dakota	64.0	84.8	74.7
Ohio	50.1	80.4	69.3
Oklahoma	52.1	80.2	72.3
Oregon	77.5	85.4	70.7
Pennsylvania	53.0	77.8	67.0
Rhode Island	52.7	88.6	76.6
South Carolina	45.5	88.1	68.1
South Dakota	70.3	87.8	69.0
Tennessee	56.6	73.8	55.6
Texas	68.9	84.5	70.5
Utah	76.9	86.2	68.7
Vermont	61.6	85.5	84.7
Virginia	51.0	76.4	69.3
Washington	78.9	89.9	77.3
West Virginia	32.0	79.8	70.7
Wisconsin	63.1	78.8	71.3
Wyoming	61.0	80.4	67.8

^a^ The prevalence of smoke-free home rules was measured at the household level. A smoke-free household is a single-parent household in which the parent reported a smoke-free home rule or a 2-parent household in which at least 1 parent reported smoke-free home rules.

^b^ The prevalence of supportive attitudes toward smoke-free rules in cars and outdoor children’s play areas was measured at the individual level.
